# Bavachinin Induces Oxidative Damage in HepaRG Cells through p38/JNK MAPK Pathways

**DOI:** 10.3390/toxins10040154

**Published:** 2018-04-12

**Authors:** Shan Wang, Min Wang, Min Wang, Yu Tian, Xiao Sun, Guibo Sun, Xiaobo Sun

**Affiliations:** 1 Beijing Key Laboratory of Innovative Drug Discovery of Traditional Chinese Medicine (Natural Medicine) and Translational Medicine, Institute of Medicinal Plant Development, Chinese Academy of Medical Sciences & Peking Union Medical College, Beijing 100193, China; wsgoodluck163@163.com (S.W.); wangmin13107@163.com (M.W.); ytian@implad.ac.cn (Y.T.); xsun@implad.ac.cn (Xiao.S.); 2 Harbin University of Commerce, Harbin 150076, China; mwang@implad.ac.cn

**Keywords:** drug-induced liver injury, Bavachinin, reactive oxygen species, p38 MAPK, JNK MAPK

## Abstract

Drug-induced liver injury is one of the main causes of drug non-approval and drug withdrawal by the Food and Drug Administration (FDA). Bavachinin (BVC) is a natural product derived from the fruit of the traditional Chinese herb Fructus Psoraleae (FP). There have been reports of acute liver injury following the administration of FP and its related proprietary medicines. To explore BVC hepatotoxicity and its mechanisms, we used the HepaRG cell line. In our recent research, we showed that BVC induces HepaRG cell death, mainly via BVC-induced oxidative damage. The formation of ROS is closely related to the activation of the stress-activated kinases, JNK and p38, while SP600125 (SP, JNK inhibitor) and SB203580 (SB, p38 inhibitor) pretreatment inhibited the generation of ROS. On the other hand, N-acetylcysteine (NAC) pretreatment prevented the phosphorylation of p38 but not that of JNK. Taken together, these data reveal that BVC induces HepaRG cell death via ROS and the JNK/p38 signaling pathways.

## 1. Introduction

Drug-induced liver injury (DILI) is a major challenge for clinicians, the pharmaceutical industry and regulatory agencies worldwide, including the FDA [1]. DILI typically manifests in a very small subset of the population under treatment with no clear dose relationship and is therefore termed an idiosyncratic event [2]. The major challenge is that, for many compounds that cause human DILI, the underlying mechanisms are not yet fully understood. Many risk factors contribute to the occurrence of DILI, such as reactive metabolites, mitochondrial toxicity, host immune-response pathways and biliary transporters [3,4,5]. On the other hand, its idiosyncratic character makes predicting DILI in preclinical tests very challenging [6]. Traditionally, in vitro systems used to address DILI have consisted of HepG2 or Huh7. In recent decades, primary hepatocyte models have evolved as the gold standard to address drug safety-related questions in vitro due to their superior functionality and liver-like phenotype. The HepaRG cell line was first derived from a liver tumor in a female patient suffering from liver carcinoma and hepatitis C infection. Some of its properties are a high degree of differentiation when cultured under certain conditions for a long time, which is similar to liver progenitors, and that it can be transdifferentiated into hepatocytes and cholangiocarcinoma cells [7]. It is different from HepG2 cell line, which is a human hepatocellular carcinoma cell line that expresses weakly functional proteins. HepaRG cells express various I Phase metabolizing enzymes (CYP1A2, 2B6, 2C9, 2E1, and 3A4), II Phase metabolic enzyme UGT, and III Phase transporter MRP, which is similar to human primary hepatocytes in functional protein expression. Moreover, HepaRG cells can maintain functional protein expression and cell viability for up to one month. Therefore, the HepaRG cell line is even superior to human primary hepatocytes for in vitro screening of chronic accumulation-induced liver injury drugs [8,9]. Wu and colleague compared HepaRG with L-02, HepG2 and hiHeps cell lines for the assessment of drug-induced liver injury and found that the HepaRG cell line is the superior in vitro model compared to the others [10]. Therefore, we chose the HepaRG cell line as our in vitro model to evaluate the liver damage caused by bavachinin in our research.

Fructus Psoraleae (FP, Bu-gu-zhi), one of the most popular traditional Chinese medicines, is composed of the dried ripe seeds of *Psoralea corylifolia* L. (Leguminosae) and has been widely used for the treatment of many conditions, including vitiligo, bone fractures, osteoporosis, etc. [11,12]. However, several cases of markedly elevated bilirubin and acute liver injury following administration of FP and its related proprietary medicines have been reported [13]. Bavachinin (BVC), a natural product from FP, has been reported to have several pharmacological activities. It has been reported as a natural pan-PPAR agonist to lower glucose and triacylglycerol levels in db/db mice [14,15]. Bavachinin, as a potent anti-asthma drug, significantly inhibits Th2 cytokine production, including IL-4, IL-5 and IL-13 [16,17]. Bavachinin has potent anti-angiogenic and anti-tumor activity in vitro and in vivo by targeting hypoxia-inducible factor-1α [18]. Some other researchers have identified that it inhibited UDP-glucuronosyltransferase 1A [19,20]. The cytotoxic effects of BVC have also been studied in various cancer cell lines [21]. However, its potential to damage the liver has not been reported, so we designed this research to explore its activity against hepatocytes. In our research, we chose HepaRG cell line to explore its hepatotoxicity, which is similar, or even superior, to human primary hepatocytes for DILI.

Reactive oxygen species (ROS) comprise one of the most commonly invoked cell death mechanisms during organ injury, including drug-induced liver damage [5]. Mitogen-activated protein kinases (MAPKs) are essential for the regulation of cell survival, the immune response, or cell differentiation upon facing endogenous or exogenous stimuli. The family of MAPKs consists of three major groups, namely the c-jun N-terminal kinases (JNKs), the extracellular regulated kinases (ERKs), and the p38 MAPKs [22]. The ERK1/2 pathway is usually linked to cell proliferation and cell survival. The stress-response MAPKs, such as JNK and p38, mostly regulate stress-induced apoptosis but are also associated with differentiation and immune responses [23]. In our research, we mainly focused on the stress-response MAPKs, JNK and p38. JNK, one of the death signaling pathways, has been reported as being involved in APAP-induced liver injury [24], plays an important role in the stress response and can be activated by various stressors, including ROS [25].

## 2. Results

### 2.1. Cytotoxicity of Bavachinin Administered to HepaRG Cell Line

**Figure 1:**
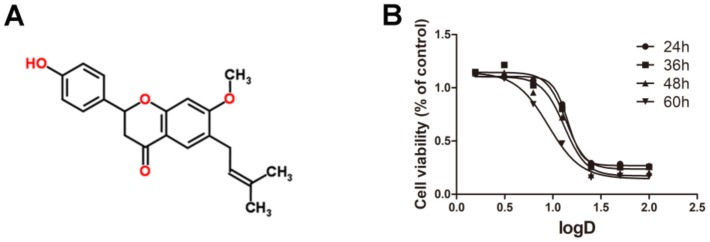
The cytotoxic effects of Bavachinin administered to HepaRG cell line: (**A**) structure of BVC; and (**B**) the dose–effect curves of BVC in HepaRG after 24, 36, 48 and 60 h treatment.

The chemical structure of BVC is shown in Figure 1A. To investigate the cytotoxic effects of various concentrations of BVC on the HepaRG cell line, we performed MTT assays after 24, 36, 48 and 60 h of BVC treatment, and the results showed that BVC inhibited cell viability in a time- and dose-dependent manner (Figure 1B). The dose–effect equations of BVC at different times are displayed in Table 1. The results showed that the 24 h BVC IC_50_ values for HepaRG cells, the concentrations at which BVC reduced HepaRG cell viability by 50% over 24 h, was 14.28 μM. Based on these results, we choose 6.25, 12.5, and 25 μM as the concentrations for further research on the mechanisms of action of BVC.

### 2.2. BVC Induced HepaRG Oxidative Stress after 24 h of Treatment

**Figure 2:**
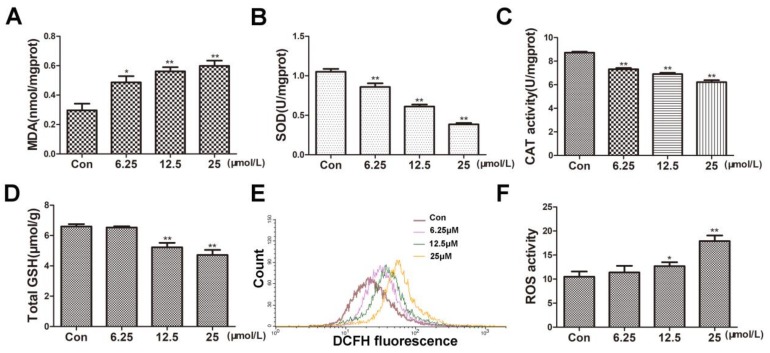
BVC increased MDA and ROS but decreased the levels of SOD, CAT, total GSH after 24 h exposure. (**A**) MDA content was measured as described before after BVC incubation. (**B**) SOD levels were detected as before. (**C**) CAT level changes are shown. (**D**) Levels of total GSH in HepaRG cells after BVC incubation are shown. (**E**) Intracellular formation of ROS after incubation with BVC in HepaRG cells in the DCF assay is shown. (**F**) Quantitative analysis of the ROS is shown. Compared with control: * *p* < 0.05 and ** *p* < 0.01.

Oxidative stress is one of the main causes of hepatocyte injury and cell death. To investigate whether BVC can induce oxidative damage in HepaRG cells, we firstly determined the oxidative damage after 24 h BVC exposure. Excessive production of reactive oxygen species (ROS) has been shown to disturb intracellular redox status homeostasis and to induce cell apoptosis or death. Malondialdehyde levels were used as surrogate assays for membrane lipid oxidation, which is one of the primary events in oxidative damage. As shown in Figure 2A, the MDA levels significantly increased in a dose-dependent manner after 24 h of incubation with 6.25, 12.5, and 25 μM BVC. Under physiological conditions, reactive oxygen species (ROS) are rapidly removed by antioxidant enzymes, including superoxide dismutases (SODs), catalase (CAT), and glutathione peroxidases (GPxs), which depend on the level of glutathione [26]. To further explore whether BVC could induce oxidative stress by damaging the antioxidant systems, we measured the changes in the levels of SOD, CAT, total GSH and ROS. In our study, the levels of both SOD (Figure 2B) and CAT (Figure 2C) significantly decreased in a dose-dependent manner after 24 h of incubation with 6.25, 12.5, and 25 μM BVC, and the level of GSH (Figure 2D) also decreased after 24 h incubation with the higher BVC concentrations (12.5 and 25 μM). The intracellular level ROS was evaluated by DCF assay after 24 h of incubation with 6.25, 12.5, and 25 μM BVC. As shown in Figure 2E,F, ROS generation significantly increased after 24 h of BVC exposure at concentrations of 12.5 and 25 μM. Taken together, these results suggested that BVC can induce marked oxidative damage after 24 h of treatment.

### 2.3. Time-Course of ROS Accumulation and p38/JNK Activation after BVC Treatment

**Figure 3:**
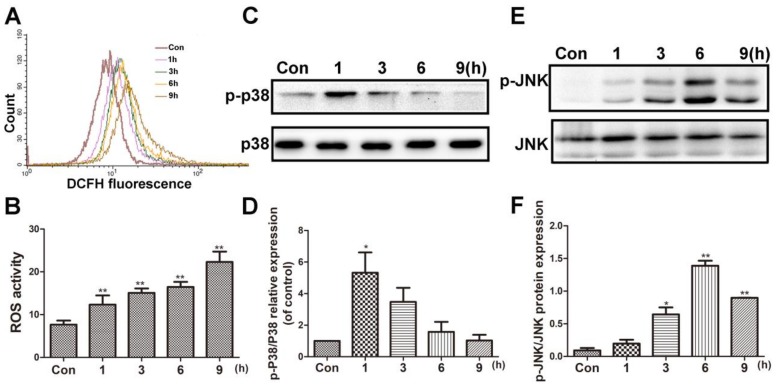
BVC induced the ROS accumulation and p38/JNK activation in time-dependent manner. (**A**) Cells were treated with 12.5 µM BVC for 1–9 h and ROS level was detected by flow cytometer analysis. (**B**) Quantitative analysis of the ROS. (**C**) Effects of BVC on p38/p-p38 expressions in HepaRG cells. The cells were treated with 12.5 µM BVC for 1, 3, 6, and 9 h, and then the expressions of the indicated proteins were evaluated by Western blotting. (**D**) Quantitative analysis of the p-p38 protein expression. (**E**) Representative Western blotting of JNK/p-JNK expressions. (**F**) Quantitative analysis of the p-JNK/JNK protein expression. Significances refer to comparison to control, * *p* < 0.05 and ** *p* < 0.01.

BVC can induce oxidative damage in HepaRG cells after 24 h of treatment. To investigate the time-course of ROS generation, flow cytometer analysis was used after 12.5 µM BVC-treated HepaRG cells at different time points (1, 3, 6, and 9 h). The result is shown in Figure 3A,B, and it demonstrated that the level of ROS was increased 1 h after exposure and continued to increase in a time-dependent manner up to 9 h after exposure. c-Jun N-terminal kinase (JNK) and p38 play critical roles in regulating stress-induced apoptosis during DILI [27,28]. To determine the effects of BVC on the JNK/p38 pathway, we examined the effects of BVC on the total expression and phosphorylation of different MAPK subgroups at different time points (1, 3, 6, and 9 h). As shown in Figure 3C,D, the phosphorylation of p38 peaked at 1 h and then declined at 3, 6, and 9 h; however, the phosphorylation of JNK (Figure 3E,F) increased in a time-dependent manner during the early stage (1, 3, and 6 h), peaked at 6 h, and then decreased at 9 h. These results revealed that p38 and JNK are both activated after the HepaRG cell line is exposed to BVC. p38 was mainly involved in the early stage (0–1 h), and JNK was involved in the later phase (1–9 h). ROS levels were sustained during this time-course. Taken together, these results demonstrated that the phosphorylation of JNK and p38 and the generation of ROS may exert synergistic effects on BVC-induced HepaRG cell damage.

### 2.4. Inhibition of p38/JNK Phosphorylation Decreased BVC-Induced Generation of ROS

**Figure 4:**
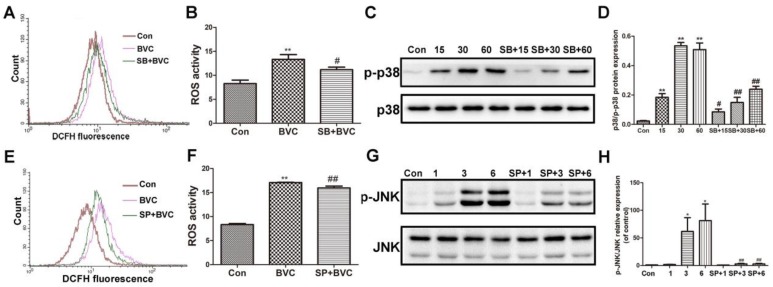
Inhibition of p38/JNK phosphorylation decreased BVC-induced ROS. (**A**,**B**) Cells were pretreated with SB203580 (a p38 inhibitor, 12.5 μM) for 2 h, followed by exposure to BVC (12.5 μM) for 1 h, and ROS were assayed by FACS. (**C**,**D**) Western blotting was used to identify its protein expression of p-p38 and p38 after BVC (12.5 μM) exposure for 15, 30, and 60 min. (**E**,**F**) Cells were pretreated with SP600125 (a JNK inhibitor, 12.5 μM) for 2 h, followed by exposure to BVC (12.5 μM) for 6 h, and ROS were assayed by FACS. (**G**,**H**) Western blotting was used to identify its protein expression of p-JNK and JNK after BVC (12.5 μM) exposure for 1, 3, and 6 h. Significances refer to comparison to control, * *p* < 0.05 and ** *p* < 0.01. **^#^** *p* < 0.05 and **^##^** *p* < 0.01 between BVC group.

The above results have demonstrated that BVC can induce ROS generation and accumulation for up to 9 h, or even up to 24 h. p38 was activated for the first hour, and JNK was activated during Hours 3–9, indicating that they were both involved the progress at different stages. Many studies have revealed that the JNK/p38 signaling pathway participates in the generation of ROS. To determine if JNK and p38 are involved in BVC-induced generation of ROS, we further investigated the impacts of SP600125 (a JNK inhibitor) and SB203580 (a p38 inhibitor) on MAPK and ROS [29,30]. First, cells were cultured in the presence or absence of SB (12.5 μM) for 2 h and then exposed to BVC for different additional amount of time. As shown in Figure 4A,B, pretreatment with the p38 signaling inhibitor SB significantly down regulated the level of ROS after 1 h of incubation with BVC. The protein levels were detected by Western blotting (Figure 4C,D) and showed that SB pretreatment clearly decreases the phosphorylation of p38 after BVC incubation for 15, 30, and 60 min. These results were consistent and confirmed that BVC induces ROS generation via the activation of p38. The relationship between the generation of ROS and JNK was also confirmed by SP. SP (12.5 μM) was used as a pretreatment for 2 h, followed by BVC incubation for 6 h, which was associated with the peak time of JNK phosphorylation. As shown in Figure 4E,F, SP pretreatment resulted in a marked decrease in the level of ROS, which was consistent with the decreased level of JNK phosphorylation (Figure 4G,H). Taken together, these results imply that BVC-induced ROS generation may be mainly attributed to the activation of the JNK and p38 pathways.

### 2.5. Inhibition of ROS Had No Effect on JNK But Disturbed the Activation of p38

**Figure 5:**
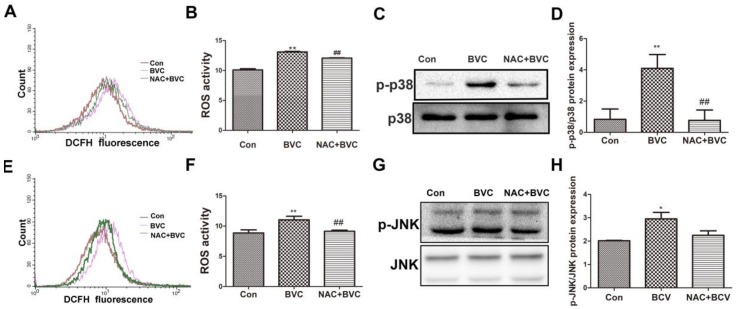
Inhibition of BVC-induced ROS via NAC had no effect on JNK but disturbed the activation of p38. (**A**,**B**) Cells were pretreated with/without NAC (20 mM) for 0.5 h, followed by exposure to BVC (12.5 μM) for 0.5 h. Intracellular ROS were measured by DCF fluorescence after DCFH-DA staining using flow cytometer. (**C**,**D**) Expression levels of p-p38 and p38 proteins were detected by Western blotting. (**E**,**F**) Cells were pretreated with/without NAC (20 mM) for 0.5 h, followed by exposure to BVC (12.5 μM) for 3 h. ROS were assayed by FACS after BVC treatment for 3 h. (**G**,**H**) Western blotting was used to identify its protein expression of p-JNK and JNK after BVC (12.5 μM) exposure for 3 h. Values were means ± SD for at least three independent experiments performed in triplicate. * *p* < 0.05 and ** *p* < 0.01 versus control. Compared to indicated groups, and **^##^** *p* < 0.01.

JNK/p38 amplification loops, which we refer to as p-JNK/p-p38-ROS-p-JNK/p-p38, have been elucidated in liver models. To evaluate whether increases in oxidative stress play a role in the activation of JNK/p38 after BVC treatment, the ability of the antioxidant NAC to protect against the BVC-induced increase in ROS was determined. HepaRG cells were treated with the antioxidant NAC (20 mM) for 0.5 h and then 12.5 μM BVC was added for different lengths of time (0.5 and 3 h) to test the protective effect of NAC against the generation of ROS [31]. From the results, BVC-induced ROS was partially prevented by NAC at 0.5 h (Figure 5A,B). The phosphorylation of p38 after NAC treatment was also detected by Western blotting (Figure 5C,D). NAC-mediated inhibition of ROS production significantly attenuated the activation of p38. Overall, these results demonstrated that there exists a p-p38-ROS-p-p38 loop after the early phase (0–1 h) of BVC (12.5 μM) treatment. The JNK loop was also explored and it was discovered that NAC could inhibit the generation of ROS at 3 h (Figure 5E,F); however, the protein level determined by Western blotting showed that there was no inhibition of JNK phosphorylation concomitant with the ROS inhibition (Figure 5G,H). Taken together, these results suggest that BVC-induced ROS generation in HepaRG cells can be reversed by NAC and that the activation of p38 is related to the generation of ROS while that of JNK is not.

## 3. Discussion

The present study demonstrated that BVC induces widespread HepaRG cell death in a dose- and time-dependent manner. BVC can induce ROS formation and damage the balance between oxidative stress and antioxidants in HepaRG cells. The time-course of ROS generation revealed that BVC-induced ROS can accumulate over time and might be the main factor inducing cell death. The stress-activated signaling pathways, JNK and p38, were activated after BVC exposure, and our data also revealed that these pathways were involved the formation of ROS. Inhibition of both pathways down regulated the production of ROS, and, furthermore, the data showed that both pathways participated in this phenomenon at different points in the time course. Additionally, it was demonstrated that ROS participate in the activation of p38 but had no effect on JNK.

Fructus Psoraleae (FP) is one of the most popular TCM and officially listed in the Chinese Pharmacopoeia. BVC is one of the ethanol extracts of FP and its content was reported to be approximately 0.032% [18], so it is not the main compound. In our research, we identified its IC_50_ on HepaRG cells was 14.28 μM, however, we did not explore its hepatotoxicity in animal models. There are many compounds in FP, and we studied one potential material basis of FP in liver toxicity in this paper and gave some guidance for further researches. However, it is not sufficient to suggest the clinicians how to avoid acute liver injury via limit the of dried ripe seeds of *Psoralea corylifolia* L. (Leguminosae), there need more animal results to confirm its hepatotoxicity in animal even human beings.

The vital roles of ROS in cellular damage have been widely investigated, and it has been suggested that ROS constitute one of the compound-dependent mechanisms underlying DILI [32]. ROS include superoxide anion radical (O2^−^), hydrogen peroxide (H_2_O_2_), hydroxyl radical (·OH) and peroxynitrite (NO), which is the main source of oxidative stress [33]. The most relevant sources of ROS are electron leaks from the mitochondrial electron transport chain, so the formation of ROS also indicates damage to the mitochondria. Mitochondrial dysfunction and oxidant stress as mechanisms of toxicity have been evaluated for several hepatotoxic drugs [5,26,34]. Increased ROS directly injure DNA, proteins, and lipids and promote cell death through the activation of stress-related signaling pathways such as those involving JNK or p38 mitogen-activated protein kinase [35]. Our data indicated that the ROS generation induced by BVC began at 30 min and extended for 24 h, perhaps involving the whole process of cell death. The underlying mechanism causing cell death is not clear.

The interaction between ROS and MAPKs, especially JNK and p38, has been thoroughly explored. In our research, we further elucidated their relationship. BVC can induce increases in JNK and p38 MAPK phosphorylation in HepaRG cells; however, they follow different time-courses after BVC exposure. p38 was activated during the early stage (0–1 h) and reached its peak at 30 min. The p38 MAPK inhibitor (SB) partially attenuated BVC-induced HepaRG cell ROS generation at 1 h, which indicated that p38 participates in BVC-induced ROS generation. On the other hand, NAC attenuated ROS generation after 30 min of BVC exposure and could down regulate p38 MAPK phosphorylation levels at the same time point. Thus, these data showed that there exists a p38-ROS-p38 loop after early treatment with BVC (0–1 h).

JNK can be activated during the late stage (3–9 h) after BVC exposure and peaks at 6 h. Furthermore, our results also showed that the indicated JNK inhibitor (SP) significantly attenuated BVC-induced HepaRG cell ROS generation at 6 h; thus, these data indicate that JNK is involved in the BVC-induced generation of ROS. NAC can inhibit the up regulation of ROS after BVC administration (6 h), but the phosphorylation of JNK as not changed. Taken together, these data showed that JNK can stimulate the generation of ROS, although ROS cannot affect the activation of JNK.

In conclusion, BVC activated p38 and JNK, which were involved in different phases of the production of ROS. In the early phase, the activation of p38 stimulated the produce of ROS, while in the late phase, JNK is activated sustain the production of ROS. Thus, ROS and the JNK and p38 MAPK pathways may play important roles at different time points in mediating BVC-induced HepaRG cell injury.

## 4. Material and Methods

### 4.1. Materials

Bavachinin (BVC, purity >98%) was supplied by Shanghai Winherb Medical S&T Development (Shanghai, China). MTT (3-(4,5-dimethylthiazol-2-yl)-2,5-diphenyltetrazoliumbromide, 0973) and NAC (N-acetylcysteine) were the products of Sigma-Aldrich (St. Louis, MO, USA). The kits for determining the malondialdehyde (MDA) content and the activity of catalase (CAT) and superoxide dismutase (SOD) were obtained from Nanjing Jiancheng Institute of Biological Engineering (Nanjing, China). Glutathione assay kit (GSH) was purchased from Beyotime Biotechnology (Shanghai, China). The Image-IT^TM^ LIVE Green Reactive Oxygen Species Detection Kit was from Life technologies (San Diego, CA, USA). SP600125, SB203588, the antibody (JNK, P-JNK, p-38, p-p38) were bought from Cell Signaling Technology (Danvers, MA, USA).

### 4.2. Cell Culture and Treatment Peking Union Medical College

HepG2 cells were purchased from Peking Union Medical College (Beijing, China). The cells were cultivated in Dulbecco’s modified Eagle’s medium, supplemented with 10% (*v*/*v*) fetal bovine serum (FBS), 100 U/mL penicillin and 100 μg/mL streptomycin. HepaRG cells were obtained from Guan Dao Biotechnology (Shanghai, China) and cultured in RPMI-1640 medium supplemented with 10% (*v*/*v*) fetal bovine serum (FBS) (Gibco, Thermo Fisher Scientific, Waltham, MA, USA), 100 U/mL penicillin and 100 μg/mL streptomycin. The media and supplements were purchased from Gibco Life Technologies (Grand Island, NY, USA). The cell cultures were kept in a humidified atmosphere containing 5% CO_2_ at 37 °C. BVC was dissolved in DMSO and incubated in FBS-free complete medium with a final DMSO concentration of 1%.

### 4.3. Determination of ROS Generation

HepaRG cells were plated on 6-well plates at a density of 1 × 10^5^ cells/well and then grown at 37 °C for 24 h. Then, the cells were treated with different compounds as indicated for the experiments. After that, all media were removed and changed to serum-free media loaded with CM-H2DCFDA (Invitrogen™, Carlsbad, CA, USA), and then the cells were incubated for 1 h at 37 °C, washed twice by PBS, digested by trypsin and re-suspended in PBS for flow cytometer analysis.

### 4.4. Western Blotting

HepaRG cells were harvested after treatment with BVC as previously described. The total protein was obtained using lysis buffer, and the protein concentration was determined by BCA assay. About 50-μg protein samples were subjected to SDS-PAGE electrophoresis and transferred to NC membranes. The membranes were blocked with 5% (*w*/*v*) nonfat dried milk for 2 h and incubated with various specific primary antibodies at 4 °C overnight. The membranes were subsequently washed with TBST and incubated with the appropriate secondary antibody at room temperature for 2 h. Following washes with TBST, the protein bands were visualized using an ECL system (CW Biotech, Beijing, China) and imaged using a Bio-Rad imaging system (Bio-Rad, Hercules, CA, USA).

### 4.5. Cell Viability

Cell viability was determined using the MTT (3-(4,5-dimethylthiazol-2-yl)-2,5-diphenyl tetrazolium, Amresco, 0973) assay as previously described [36]. Briefly, HepaRG cells were plated on 96-well plates at a density of 8 × 10^4^ cells/well and then grown at 37 °C for 24 h. The cells were pretreated with BVC for different lengths of time from 24 h to 60 h. Twenty microliters of MTT (5 mg/mL) was added to each well and incubated for 4 h. The medium was removed, and the formazan crystals were dissolved with dimethyl sulfoxide (DMSO). The absorbance was measured at 570 nm on a microplate reader (TECAN Infinite M1000, Grödig, Austria).

### 4.6. Measurement of MDA, CAT, GSH, SOD

HepaRG cells were harvested after treated 24 h by BVC, then were measured the activities of superoxide dismutase (SOD), catalase (CAT), and the content of malondialdehyde (MDA) and glutathione (GSH) by following the manufacturer’s instructions.

### 4.7. Statistics

All data are expressed as the means ± SD of at least three independent experiments. Significant differences between the groups were determined by ANOVA. Data analyses were performed using GraphPad Prism 5 (GraphPad Prism Software, 2012, La Jolla, CA, USA).

## Figures and Tables

**Table 1:** BVC dose–effect equation in HepaRG.

Time (h)	Equation	IC_50_ (μM)	R^2^
24	*y* = 0.2698 + 0.8362/(1 + 10^(−6.7567+5.850*X*)^)	14.28	0.9976
36	*y* = 0.2364 + 0.9076/(1 + 10^(−4.9607+4.363*X*)^)	13.71	0.9858
48	*y* = 0.1730 + 0.9310/(1 + 10^(−2.2432+3.792*X*)^)	13.15	0.9837
60	*y* = 0.1457 + 0.9993/(1 + 10^(−2.4425+2.563*X*)^)	8.975	0.9956

## Abbreviations

DILI	Drug-induced liver injury
BVC	Bavachinin
MDA	Malondialdehyde
ROS	Reactive oxygen species
SOD	Superoxide dismutase
GSH	Glutathione
CAT	Catalase
MTT	3-(4,5-dimethylthiazol-2yl)-2,5-diphenyltetrazolium bromide
MAPK	Mitogen-activated protein kinases
JNK	c-jun N-terminal kinases
SP	SP600125
SB	SB203580
NAC	N-acetylcysteine
FP	Fructus Psoraleae
